# "Ready, set, go" - are we facilitating change in the (pre-) contemplative clinician?

**DOI:** 10.1186/2050-2974-2-S1-O21

**Published:** 2014-11-24

**Authors:** Ulrike O'Sullivan, Julie McCormack, Matthew Hamilton

**Affiliations:** 1 Specialised Child and Adolescent Mental Health Services, Princess Margaret Hospital for Children, Perth, Australia

## 

Health professionals in non-specialist treatment settings often feel ill equipped to provide treatment for and manage clients with eating disorders and therefore can be reluctant (pre-contemplative) to provide services. Consequently, health providers willing and able to see clients with eating disorders can be sparse in the community.

The Eating Disorders Training and Evaluation Centre (EDTEC) provides state-wide training to health professionals about eating disorders in WA. EDTEC has a mandate to improve access to treatment for those affected by eating disorders by improving awareness, and the understanding, knowledge and skills necessary for appropriate and effective recognition and treatment of eating disorders.

For over three years, EDTEC has collected direct feedback from approximately 1000 training workshop participants, with self-reported increase in knowledge, competence and confidence in treating clients with eating disorders. It is acknowledged this method of evaluation does not provide an insight into whether the clinician undergoing training actually improves their knowledge and skills and in terms of community access, there remains the question of whether training really leads to a recognisable increase in service provision.

This paper will report on a longer term follow up, where EDTEC sought further information via an online survey of those who have attended training over three years. The main aim of the survey was to establish the post-training impact on professionals seeing clients with eating disorders and the factors involved in increased service provision. Future directions will be discussed, including additional methods of evaluation and innovative methods for supporting the community clinician.

This abstract was presented in the **Service Initiatives** stream of the 2014 ANZAED Conference.

